# Clinical evidence and biological mechanisms linking obesity to adverse outcomes in pediatric and adolescent acute lymphoblastic leukemia

**DOI:** 10.3389/fonc.2026.1918955

**Published:** 2026-09-04

**Authors:** Cate Cheney, Elyse Page, David Yeung, Deborah White

**Affiliations:** 1 Targeted Leuakemia Research Group, South Australian Health and Medical Research Institute, Adelaide, SA, Australia; 2 College of Health, Adelaide University, Adelaide, SA, Australia; 3 Department of Haematology, The Royal Adelaide Hospital, Adelaide, SA, Australia; 4 Australian and New Zealand Children’s Oncology Group (ANZCHOG), Clayton, VIC, Australia; 5 Australian Genomics Health Alliance (AGHA), The Murdoch Children’s Research Institute, Parkville, VIC, Australia

**Keywords:** acute lymphoblastic leukemia, body mass index, metabolic dysregulation, obesity, treatment delay

## Abstract

This review critically evaluated the relationship between obesity and survival outcomes in Acute Lymphoblastic Leukemia (ALL), highlighting key evidence gaps and exploring the biological mechanisms that may contribute to this association. In patients with ALL, obesity is consistently associated with a higher incidence of severe treatment-related toxicities, increased frequency of therapy delays, and reduced event-free survival. This connection is most pronounced in pediatric and adolescent ALL populations, whereas evidence supporting a similar effect in adults remains limited. Proposed biological mechanisms underlying these adverse outcomes include increased bone marrow adiposity, chronic low-grade inflammation, dysregulated insulin signaling and obesity-associated alterations in the gut microbiome. The current body of evidence identifies obesity as a clinically significant risk factor that defines a vulnerable subgroup of ALL patients, supporting the need for enhanced toxicity surveillance, individualized treatment strategies, and optimized supportive care interventions. Elucidation of the biological pathways linking obesity to adverse ALL outcomes may also inform the development of novel therapeutic approaches aimed at mitigating obesity-driven morbidity and mortality in this high-risk population.

## Introduction

Acute Lymphoblastic Leukemia (ALL) is an aggressive hematological malignancy with a rising global prevalence (1). Global incidence increased by approximately 5.7% between 1990 and 2021, with the greatest burden observed among pediatric populations (2). Despite significant increases in survival rates, driven by more expedient diagnosis, genomic subclassification, and availability of novel therapeutics (1), additional novel interventions are needed to improve long-term remission rates and minimize late effects (3). Concurrently, the global prevalence of obesity has increased substantially from 1990 to 2022, with over 35 million children under the age of 5 identified as overweight and 160 million children and adolescents aged 5–19 living with obesity (4, 5). Given the rising global prevalence of both obesity and ALL, an expanding body of epidemiological evidence indicates a clinically meaningful association between obesity and adverse ALL outcomes. This review synthesizes the current literature examining this relationship and explores the proposed biological mechanisms through which obesity may contribute to increased morbidity and mortality in ALL.

The standardized diagnosis of overweight/obesity is determined by measuring an individual’s height and weight to calculate Body Mass Index (BMI). The World Health Organization (WHO) categorize overweight as a BMI greater than or equal to 25 kg/m (2), and obesity as a BMI greater than or equal to 30 kg/m (6). WHO consensus initially defined ethnicity-specific BMI cutoffs in South Asian and Chinese populations, though subsequently settled on ethnicity agnostic definitions (7). Nonetheless, increasingly robust data, the WHO and the UK National Institute for Health and Care Excellence (NICE) identify adjusted BMI values for a subset of ethnic populations, with obesity classified as 27.5 kg/m (2) or above in South East Asian populations (8). In children obesity is defined as a weight-for-height ratio > 3 standard deviations (SD) above the ethnicity specific WHO Child Growth Standards median for those young than 5 years, and defined as >2 (9). In children aged 5 to 19 years, obesity is defined as >2 SD in those aged 5–19 years (9). Since 1975, the global burden of obesity has increased tenfold in the pediatric and adolescent population (10). Obesity prevalence is highest in high-income countries; however, elevated age-standardized mean BMI values for children and adolescents are observed in Melanesia, Latin America, and the Caribbean (10).

Obesity is a well-established risk factor for increased all-cause mortality and multimorbidity across numerous diseases (11). In a previous oncology-specific review, Petrelli et al. suggested that obesity is associated with a 17% increased risk of cancer-specific mortality and a 13% risk of cancer recurrence (12). Historically, the correlation between obesity and poor oncological outcomes has been attributed to the disruption of drug pharmacokinetics (13) and underdosing of targeted therapies (14). Reviewed by Gouju and Legeay in 2023 relevant factors include decreased drug absorption, altered metabolism, increased distribution volume to weight of lipophilic drugs and decreased distribution volume to weight of hydrophilic drugs (15). Despite research in solid tumors, mechanistic studies examining causal links between adipocyte-rich microenvironments and hematological malignancies are still scarce.

Suspected drug sequestration and altered pharmacokinetics in adipose tissue both highlight the importance of body weight dosing. In hematological oncology, chemotherapy is often dose-adjusted in obese patients [an example being widely practiced is dose reductions for hematopoietic cell transplant conditioning regimens (16)]. As an example of particular relevance in ALL, asparaginase is often dose-reduced in obese patients, who have a higher incidence of hepatic steatosis, known to decrease drug metabolism and increase toxicity (17). Immuno- and targeted therapies, increasingly used in many cancers, are usually dosed irrespective of weight. It is uncertain whether such schedules are optimal in all circumstances.

This review aims to critically appraise clinical evidence linking obesity and adverse ALL outcomes and summarize emerging biological mechanisms for this association.

## Methodology

**Figure 1:**
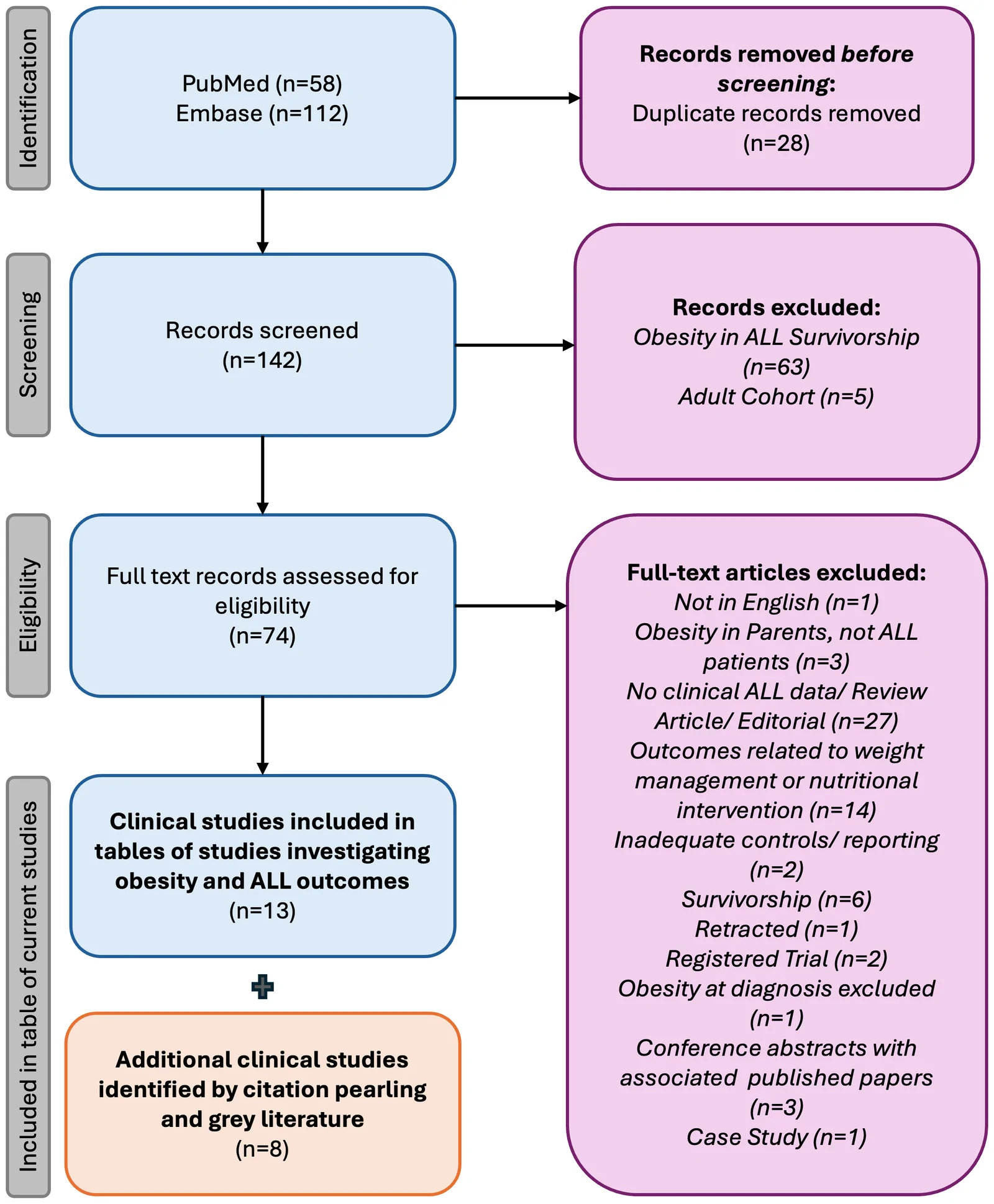
PRISMA flow diagram illustrating the identification, screening and inclusion of clinical studies discussed in this review.

Clinical studies were identified through structured searches of PubMed and EMBASE from the inception of the review in May 2025 to revisions in July 2026. The following search strings were utilized; PubMed Search String: ((Acute Lymphoblastic Leukemia[MeSH Major Topic]) AND (obesity[MeSH Major Topic]) AND (y_10[Filter])) OR ((Acute Lymphoblastic Leukaemia[MeSH Major Topic]) AND (pediatric obesity[MeSH Major Topic]) AND (y_10[Filter])), EMBASE Search String: Acute Lymphoblastic Leuakemia/and Obesity.m_titl. Limit to yr=“2015-Current”. Records were screened by title and abstract, followed by full-text assessment against pre-defined inclusion criteria (**Figure 1**). Additional clinical studies were identified by citation pearling of included articles, relevant reviews and grey literature (conference abstracts). As a narrative review, the search and selection process of clinical studies was intended to be comprehensive but not conducted according to systematic review standards. The pre-clinical evidence base exploring mechanistic connections between obesity and ALL was assembled through a targeted web-based search of the published literature.

## Associations between obesity at diagnosis and clinical outcomes

### Pediatric cohort

Overall survival rates for Pediatric ALL cohorts now exceed 90%; however, treatment is associated with severe dose-limiting side effects and significant clinical sequelae across the life course (18). Here, we summarize studies investigating clinical connections between obesity and treatment outcomes in children diagnosed with ALL (**Table 1**).

**Table 1:** Studies investigating correlations between obesity/nutritional status and acute lymphoblastic leukemia (ALL) in pediatric patients.

Study	N=	Disease stratification	Study design	Weight/nutritional status stratification	Median age (range)	Treatment protocol	Study location/ethnicity (self-reported)
(19) (Saidyk, 2020)	49	n/a	RCS	**Healthy** (BMI 5% -85%ile) **Obese** (BMI >95%ile)	Age not reported as a covariate due to small sample numbers	AALL0232, AALL0331, AALL08P1, AALL0932	USA/ Caucasian
(20) (Wadhwa, 2023)	647	**B-lineage:** 594 **T-lineage:** 75 **HR:** 282	RCS	**Underweight/Normal** (BMI<45%ile) **Overweight/Obese** (BMI = 85%-98%ile) **Extreme Obesity** (BMI>99%ile)	5 (1-18)	AALL03N1	USA/ Non-Hispanic White: 225 Hispanic: 225 African America: 120 Asian: 106
(21) (Egnell, 2021)	1443	**B-lineage:** 1312 **T-lineage:** 131	RCS	**Underweight** (BMI<17 kg/m^2^ **Healthy Weight** (BMI 17-<25 kg/m^2^) **Overweight** (BMI 25-<30 kg/m^2^) **Obese** (BMI >30 kg/m^2^)	4.7 (2-17.9)	NOPHO ALL2008	Nordic countries, Estonia, Lithuania
(22) (Meenan, 2019)	155	**B-lineage:** 135 **T-lineage:** 20	RCS	**Obesity** (BMI >95%ile for age and sex)	6 (2-22)	Not specified	USA/ Caucasian: 139 Black-American: 12 Indian: 2 Asian: 1
(23) (Prasca, 2018)	294	**B-lineage SR:**137 **B-lineage HR:** 132 **T-Lineage:** 25	RCS	**Normal** (BMI <85%ile) **Overweight** (BMI 85-94.5%ile) **Obese** (BMI >95%ile)	7 (n/a)	CCG1991, AALL0331, AALL0932, modified CCG1961, AALL08P1, AALL0232, AALL1131, AALL0434	USA/ Non-Hispanic: 68 Hispanic: 226
(24) (Park, 2023)	62	**B-Lineage** **T-Lineage** **Mixed Phenotype** ***Numbers not reported***	Multi-Centre Study PCS	**Normal** (BMI <85%ile) **Overweight** (BMI 85-94.5%ile) **Obese** (BMI >95%ile)	8 (5-21)	n/a	USA/ White: 33 Black: 11 Asian: 2 Other: 16
(6) (Panetta, 2025)	564	**n/a**	RCS	**n/a**	n/a	St Jude Children’s Research Hospital Total XVI protocol study	USA/ Hispanic: 36 Non-Hispanic: 503 Other: 22
(25) (Orgel, 2014)	198	**BP-ALL**	RCS	**Lean** (BMI <85%ile) **Overweight** (BMI 85-94.5%ile) **Obese** (BMI >95%ile)	8.2 (1.2-18.9)	COG; SR-ALL: CCG-1991, AALL0331, AALL0932; HR-ALL: CCG-1961, AALL08P1, AALL0232, AALL1131	Hispanic: 157 Non-Hispanic: 41
(26) (Mittelman, 2021)	70	**B-ALL**	Analysis of data from 3 PCS	**Obese** (BMI >95%ile) **Obese** (BMI >30 kg/m^2^)	15.2	n/a	Hispanic: 16 Non-Hispanic: 54
(27) (Puttaswamy, 2024)	39 (ALL) 39 Controls	**ALL and Matched controls**	Crosss-Sectional Study	Various Anthropometric characteristics	4.6 (2.7-6.5)	n/a	n/a
(28) (Ghosh, 2020)	4762	**B-ALL / T-ALL**	RCS	**Underweight** (BMI<5%ile) **Normal** (BMI 5-85%ile) **Overweight** (BMI 85-94.5%ile) **Obese** (BMI >95%ile)	11.3 (1-30)	AALL0232 AALL0434 AALL0622 AALL074P AALL1131	White: 2750 Black: 419 Hispanic: 1140 Unknown: 417
(29) Ladas, 2025	794	**B-ALL: 697** **T-ALL: 97**	PCS	**BMI *z* score** **Overweight (>1.4)** **Obesity (>1.65)**	6.7 (1.0-17.9)	DFCI 05-001	Hispanic: 136 Non-Hispanic: 553
(30) Núñez-Enríquez, 2019	1070	**Pre-B: 1077** **B-ALL:20** **T-ALL:114** **Biphenotypic: 42**	RCS Multicentre	**Normal** (BMI 5-85%ile) **Overweight** (BMI 85-94.5%ile) **Obese** (BMI >95%ile)	n/a	Protocol varied based on treating hospital	n/a
(31)(Zarrabi, 2025)	66	**Ph-Like B-ALL**	RCS	**Obese** (BMI >95%ile) – Patients ≤19 yrs *Or* (BMI 30 ≥ kg/m^2^) – Patients >20 yrs	16.2 (2-20)	Not reported	Hispanic/ Latinx: 54 Non-Hispanic: 12
(32)(Sassine, 2023)	3458	***n/a***	RCS	**Obese** (BMI >95%ile)	2-18	Protocol varied based on treating hospital	n/a

HR, high risk; SR, Standard risk; SO, Severe Obesity; HzR, hazard ratio; OR, odds ratio; CT, clinical trial; RCS, retrospective cohort study; PCS, Prospective cohort study; RD, Refractory Disease; EMI, Extramedullary involvement.

#### Genomic and prognostic associations

Several clinical studies have examined the relationship between obesity at diagnosis and prognosis (22, 28). Retrospective analysis of American patient cohorts link pre-treatment obesity and central nervous system (CNS) involvement at diagnosis, particularly in male and Hispanic patients (28). However, the high incidence of the high-risk *CRLF2*-rearranged ALL in Hispanic patients may confound this association (33). However, obesity and genomic subtypes may themselves be correlated - *CRLF2* rearrangements tend to occur in patients with higher fat mass, body fat percentage, and BMI, using analyses adjusted for ethnicity. Notably, Mittelman *2021* reports a 13% increase in the incidence of *CRLF2*r ALL per kilogram of excess fat mass above healthy weight guidelines (11). Additional cohorts reinforce a link between high BMI and CNS involvement (22, 34), whereas a normal BMI is associated with the favorable prognostic presence of double trisomy of chromosomes 4 and 10 (28).

#### On-treatment side-effects

There is a strong correlation between on-treatment obesity and Serious Adverse Events (SAEs) reported through prospective clinical trials (21–23). SAEs, as a surrogate of severe toxicities that increase both morbidity and mortality, include any event that leads to hospitalization, or prolongation of in-patient stay and may result in death or, more commonly, therapeutic interruptions and protocol deviations. Egnell et al. analyzed results from the Nordic Society of Pediatric Haematology and Oncology ALL2008 protocol, and found obese patients have a longer median treatment delay (period from diagnosis to initiation of maintenance-1) as compared to healthy-weight children. The increased time taken to deliver induction therapy was attributed to an increased incidence of SAEs in obese children (25% incidence) compared to healthy-weight controls (11.8% incidence) (21). Across this cohort, 52.6% of patients experienced at least 1 SAE, with the prevalence increasing with age and obesity status. Metabolic and organ dysfunction, such as hyperlipidemia-associated pancreatitis, liver dysfunction and kidney dysfunction were implicated in many of the treatment delays. Specifically, obese children had an increased risk of hyperlipidemia compared to healthy-weight children, with approximately one in four obese B-ALL patients less than 10-years of age identified as >5 times the upper normal lipid limit of triglycerides (2 mmol/L) (35). Obesity also increases the risk of treatment-related diabetes. Meenan *2019* demonstrates that obese children with high-risk ALL had a 6.63 odds ratio of requiring hyperglycemic intervention (22). Obese children are also found to be at a higher risk of symptomatic venous thromboembolism (VTE) compared to healthy weight or overweight patients. Prasca *2018*, identified that obesity at diagnosis was correlated with a high risk for VTE requiring intervention during the induction phase of treatment; however, in a single institutional context, generalizability of the study is limited (23).

#### Considerations for drug dosing

Multiple studies have evaluated safe dosing of chemotherapeutic drugs in the obese patient population (6, 19, 21, 36, 37). Obesity is well characterized to modulate drug pharmacokinetics: volume of distribution, clearance, protein binding, and absorption (38). Additionally, pharmacodynamic parameters are frequently disturbed (39). Dosing of antineoplastic agents in the obese population is particularly challenging due to the balance between efficacious treatment outcomes and high-dose toxicity. Historically, dose calculation has been based on body surface area (BSA), with many chemotherapeutic agents dose-capped at a BSA of 2.0 m (2) in obese patients (40); however, identification of systemic underdosing and inferior survival in this cohort has prompted clinical transition to therapeutic dose monitoring (TDM) to inform individualized dosing (40). Facilitating this, area under the plasma concentration-time curve appears to be more closely correlated with pharmacodynamics compared to dose per unit of BSA (41). The American Society of Clinical Oncology (ASCO) recommends full, weight-based cytotoxic chemotherapy doses utilized in obese adults with cancer, limiting dose capping or use of ‘ideal’ body weight dosing (42). Additionally, they present therapeutic drug monitoring-informed dosing as a key priority for future research, with TDM associated with higher response rates in orally dosed antineoplastic agent fluorouracil (43); however, many chemotherapeutic options for ALL are under investigated in this context.

Asparaginase, a key component of ALL antineoplastic therapy, is rapidly cleared from the plasma by the liver, spleen and bone marrow-resident phagocytic cells (44). Egnell et al, demonstrates older obese children (10-17.9 yrs) are less likely to complete asparaginase treatment on time, with 57.1% of older obese children experiencing asparaginase treatment discontinuation due to toxicities (21). Panetta et al, reported that the clearance of pegaspargase is decreased in obese compared to non-obese patients during induction therapy; however, no difference in toxicity or outcomes was reported in their subsequent analysis of St. Jude’s Total XVI cohort (6). Full mechanisms of obesity-mediated decreases in asparaginase clearance are not clearly determined; however, hepatic metabolic stress is hypothesized as a key intersection between obesity and asparaginase toxicity (17). Overall, the relationship between obesity and asparaginase-associated toxicity in ALL appears formulation and age-dependent, with consistent risk observed for native asparaginase but less reproducible effects reported with pegaspargase.

Vincristine, known to cause neuropathy, is a critical component of ALL chemotherapy regimens. In one study, a mixed effects analysis demonstrates a trend for increased risk of neuropathy in obese patients, compared to their ‘healthy’ weight counterparts (19). Unfortunately, due to the small sample size, other relevant factors such as age were not reported as covariates.

A standard of pediatric and AYA ALL treatment is the administration of intravenous “high-dose” methotrexate (HDMTX, >1g/m (2)) to penetrate the CNS. Methotrexate is a highly hydrophilic therapeutic, with dosing by body weight or BSA at risk of overestimating the body’s capacity for functional elimination. A large cohort analysis of cancer patients (solid and hematological receiving BSA-adjusted dosing of high-dose methotrexate, as measured by a three-compartment pharmacokinetic model, suggested that stratified dosing outperforms BSA and flat dosing to achieve target exposure (45). In this context, stratified dosing accounts for BSA-based stratification at the lower (<25^th^) and upper (>75^th^) BSA extremes to avoid toxicity in patients with uncharacteristically deviated BSAs (45). A 2021 study demonstrated BSA dosing resulted in children with a high body fat percentage (45% ± 3%) being twice as likely as healthy-weight children to develop delayed MTX elimination, defined in that study as concentrations >0.4µmol/L at 48 hours (36). Importantly, delayed MTX can trigger acute renal injury and systemic toxicity, potentially exacerbating or underlying serious adverse events in obese ALL patients (46). More recently, a visceral fat area of 47cm (2) or greater, as determined by bioelectrical impedance analysis, has been reported to increase the risk of late MTX toxicity 6-fold in ALL patients aged 6–18 years (37). Thus, stratified dosing accounting for BSA extremes may be required as a form of obesity-modulated capped dosing to avoid potential MTX toxicity.

Tyrosine kinase inhibitors (TKI) are routinely used in Philadelphia Chromosome positive ALL(Ph+), and increasingly in Ph-like genotypes. Existing data suggest obesity negatively influences tyrosine kinase pharmacokinetics (47, 48). In Chronic Myeloid Leukemia (CML), obese patients take longer to achieve complete cytogenetic response and/or major molecular response (MMR), and have lower overall rates of MMR (47). It has been suggested that increasing imatinib dosage in morbidly obese patients may assist in achieving clinical molecular milestones (48).

Although multi-agent chemotherapy and targeted therapies have improved outcomes in ALL, allogenic hematopoietic stem cell transplantation (HSCT) is a cornerstone of curative therapy for high-risk or relapsed ALL. Obesity increases the risk of graft versus host disease (GVHD) and non-relapse mortality (NRM) in ALL patients less than 40 years of age, after adjustment for patient age, donor age, conditioning intensity, HLA compatibility, pre-transplant disease status, donor/recipient sex, and disease risk index (49). Systematic analysis of overall HSCT in AML patients reveals that obesity did not significantly affect the prognosis of patients; however, in cases of allogenic HSCT, obesity increased the risk of mortality and, conversely, also decreased the risk of relapse (50).

#### Global outcomes

Several retrospective cohort analyses have identified a correlation between obesity and poor survival in pediatric cohorts (20, 25, 30). American children, with extreme obesity, treated on AALL03N1 demonstrated a higher cumulative incidence of relapse at 4 years post-treatment initiation compared to healthy weight counterparts (20). Multivariate analysis further confirmed this relationship, with extreme obesity resulting in a 2.4-fold greater hazard of relapse compared to underweight/normal weight controls. This association between relapse and obesity was only present in extreme obesity (BMI>99% percentile); with overweight/obese patients (BMI 85%-98% percentile) experiencing similar relapse rates as controls (20). Orgel *2014*, demonstrated a significant association between obesity and MRD positivity post-induction, showing poorer event-free survival (EFS) in obese and overweight children (25). Furthermore, Núñez-Enríquez *2019*, reported that overweight and obese patients were a vulnerable population of ALL patients at higher risk of mortality on treatment (30). Dombrowski *2019*, however, failed to recapitulate previously observed remission and survival differences between obese and non-obese patients (51). Their single-center retrospective chart review observed no significant differences between remission rates or secondary outcomes (time to absolute neutrophil count, incidence of febrile neutropenia, clinical or microbiological infection, in-hospital mortality and survival at 6 months) when comparing obese versus non-obese ALL patients. Collectively, these studies suggest a severity-dependent association between obesity and adverse outcomes in pediatric ALL, with stronger effects observed in children with extreme obesity and in analyses incorporating early treatment response markers such as MRD. However, smaller single-center studies with shorter follow-up periods are limited by a lack of sufficient power to detect survival differences in these cohorts.

The rising global prevalence of childhood obesity represents a significant concern, as accumulating evidence suggests a compelling association between obesity and poorer outcomes in ALL.

### Adolescent and young adult cohort

#### Survival outcomes

BMI is consistently reported as an independent risk factor for outcome in the adolescent and young adult (AYA) cohort (**Table 2**). A BMI greater than 30 kg/m (2) is associated with inferior disease-free survival (DFS), overall survival (OS) and relapse, though underlying mechanisms remain unknown (29, 55, 57). In a 2022 Nordic retrospective study, this correlation was seen mainly in non-high-risk groups, with severely obese patients at standard or intermediate risk showing poorer EFS and higher relapse risk (52), with the effect of obesity likely eclipsed in patients with high risk of relapsed determined by genomic drivers. The Australian Leukemia and Lymphoma Group (ALLG) ALL06 trial reported a BMI ≥30 kg/m (2) is significantly associated with poorer disease-free (53.3% vs 77.5%) and overall survival (49.2% vs 81.1%) compared to BMI <30 kg/m (248). Their follow-on study ALL09, showed some improved outcomes in obese patients. This study added blinatumomab to allow for chemotherapy de-intensification and BMI-informed pegaspargase dosing, both of which may be beneficial in obese cohorts (58).

**Table 2:** Studies investigating correlations between obesity/nutritional status and acute lymphoblastic leukemia (ALL) in adolescent and young adult patients.

Study	N=	Disease stratification	Study design	Weight/nutritional status stratification		Median age (range)	Treatment protocol	Study location/ethnicity (self-reported)
(52) (Egnell, 2023)	416	**B-lineage:** 282 **T-lineage:** 134 **Non-HR:** 234 **HR:**182	RCS	**Underweight** (BMI<18.5 kg/m^2^) **Healthy Weight** (BMI 18.5-<25 kg/m^2^) **Overweight** (BMI 25-<30 kg/m^2^) **Obese** (BMI 30-<35 kg/m^2^) **Severely Obese** (BMI >35kg/m^2^)		26.6 (18.0 -45.9)	NOPHO ALL2008	Nordic countries, Estonia, Lithuania
(53) (Du, 2024)	115	**B-ALL**: 58 **DLBCL:** 57	RCS	**High Risk** BMI >24kg/m^2^ + any obesity related underlying disease	**Low Risk** None of the above risk factors	43 (29-57)	CAR-T Therapy	Hong Kong, China
(54) (Shimony, 2023)	388	**B-ALL:** 167 **T-ALL:** 60	CT	***Patients 15-20*** **Underweight** (BMI< 5%ile) **Normal** (BMI 5%-84.99%ile) **Overweight** (BMI 85%-94.99%ile) **Obese** (BMI >95%ile)	***Patients >20*** **Underweight** (BMI<18.5 kg/m^2^) **Normal** (BMI 18.5-<25 kg/m^2^) **Overweight** (BMI 25-29.99 kg/m^2^) **Obese** (BMI >30 kg/m^2^)	24 (15-50)	Pediatric 00-001 Adult 01-175 Pediatric 05-001 Adult 06-254	USA/
(55) (Greenwood, 2021)	82	**B-ALL:** 57 **T-ALL:** 23 **Unknown:** 2	CT	**Normal** BMI < 30 kg/m^2^ **Obese** (BMI >30 kg/m^2^)		22.7 (16.6 -38.8)	ALLG ALL06	Australia
(56) (Ross, 2022)	168	r/r B-ALL	RCS	**Underweight** (BMI<5%ile) **Normal** (BMI 5-85%ile) **Overweight/ obese** (BMI >85%ile)		(2-22)	n/a	Pediatric Real World CAR Consortium institutions
(Greenwood, 2026)	55	**Pre-B-ALL** (Without *BCR::ABL1* rearrangement)	CT	**Normal** BMI < 30 kg/m^2^ **Obese** (BMI >30 kg/m^2^)		25 (16-39)	ALLG ALL09	Australia

HR, high risk; SR, Standard risk; SO, Severe Obesity; HzR, hazard ratio; OR, odds ratio; CT, clinical trial; RCS, retrospective cohort study; RD, Refractory Disease; EMI, Extramedullary involvement; r/r, Relapse/Refractor.

#### On-treatment and direct drug-associated side effects

Obese patients often experience more treatment toxicity, such as higher incidences of venous thromboembolism, liver toxicity, and hyperlipidaemia on asparaginase regimens (57). Patients receiving CAR-T therapy who develop adverse reactions are also more likely to experience a severe episode if identified as obese or suffering from obesity related metabolic conditions at diagnosis (57). Specifically, hyperlipidemia is identified as having a statistically significant relationship with severe cytokine release syndrome, but it is only significant at a univariate level (53). Additionally, individual case studies highlight the occurrence of severe drug toxicities in obese ALL patients upon administration of generally well-tolerated chemotherapeutic dosing schedules. Fisher et al. reported severe toxicities in an obese 15-year-old (BMI > 99^th^ percentile) with high-risk pre-B-ALL and hepatic steatosis who developed multi-organ failure after methotrexate, underscoring the need to consider pharmacodynamics in obese children, who have a higher risk of hepatic steatosis (34% risk compared to 8% in non-obese children) (59).

### Limitations of current clinical studies

Although the clinical studies presented in this review provide important evidence correlating obesity with ALL outcomes, several limitations inhibit the interpretation of study results. Twelve of fifteen pediatric studies and half of the AYA studies available were retrospective in design, which limits causal inference and introduces potential biases related to confounding factors and inconsistent follow-up periods (60). Furthermore, studies harbored substantial heterogeneity across study populations, with variable age distribution, disease risk profile (subtype and prognosis), cohort ethnicity and definition of obesity cutoffs. Additionally, emerging research moving away from BMI as a measure of obesity will be difficult to align with historical studies that have consistently relied on BMI. Due to the global nature of the studies, there was also significant variation in treatment protocols, supportive care and outcome measures, making direct comparison between cohorts challenging. Together, these issues highlight a need for caution in interpreting the existing literature, underscoring the value of prospective trials using standardized definitions with harmonized treatment and outcome measures to determine a causal relationship between obesity at diagnosis and poor outcomes in ALL.

Additionally, Body mass index does not adequately capture true body composition or visceral adiposity and therefore may misclassify patients with similar BMI values but different fat distribution of lean mass (61). Alternative measurements of body composition include body fat percentage (BF) and visceral adiposity as measured by dual-energy X-ray absorptiometry (DXA) (62), waist-to-hip ratio (WHR) (63), and bioelectrical impedance analysis (BIA) (64). The limitations of body mass index in the pediatric ALL cohort are explored by Orgel et al., 2019, highlighting the inadequacy of BMI tracking during ALL therapy to accurately reflect changes in body composition (65). Importantly, Orgel reports a high frequency of sarcopenic obesity (gain BF % with a loss of lean muscle mass LMM) during ALL treatment, as measured by DXA, which resulted in a poor correlation between BMI changes and overall BF (65). Despite these considerations, the majority of available studies report outcomes using BMI; therefore, results in this review are presented using this measure to ensure consistency with existing literature and to allow comparison across studies.

Clinical management should incorporate proactive supportive care measures to reduce side effect burden and preserve treatment tolerance. Nutritional assessment and early dietitian referral may support maintenance of energy intake, prevent unintentional weight gain, and support the management of gastrointestinal side effects during therapy. In the pediatric ALL setting, the adaptation of family-based health promotion strategies is emerging, with trials increasingly focused on preventing weight gain during treatment. A 2021 clinical trial assessed the feasibility of a dietary intervention designed to minimize weight gain during ALL induction therapy and reported strong feasibility, although a larger cohort is needed to confirm efficacy (66). During maintenance therapy, a three-visit nutritional intervention has been shown to attenuate weight gain, as measured by BMI z-score; however, this finding was derived from a retrospective study and should be validated in a prospective cohort (67). More recently, the NOURISH-ALL (NCT06050850) trial, informed by the Obesity-Related Behavioural Intervention Trials model phase 1a, is integrating evidence-based nutrition and physical activity recommendations within the current interventional framework for weight management in ALL survivors, NOURISH-T (68). This study aims to promote positive health behaviors despite the complexity of ALL treatment and is currently pre-recruitment, with primary completion expected in 2028. Regular metabolic monitoring, including weight, liver function, blood glucose and other treatment-specific laboratory parameters, is already standard during ALL therapy to enable early identification and triage of serious adverse events. In patients who are overweight or obese at diagnosis, escalation to endocrinology consultation may also be considered to facilitate early identification and management of thyroid dysfunction, adrenal insufficiency and hyperglycemia.

#### Potential biological mechanisms mediating ALL pathophysiology and on-treatment complications

**Figure 2:**
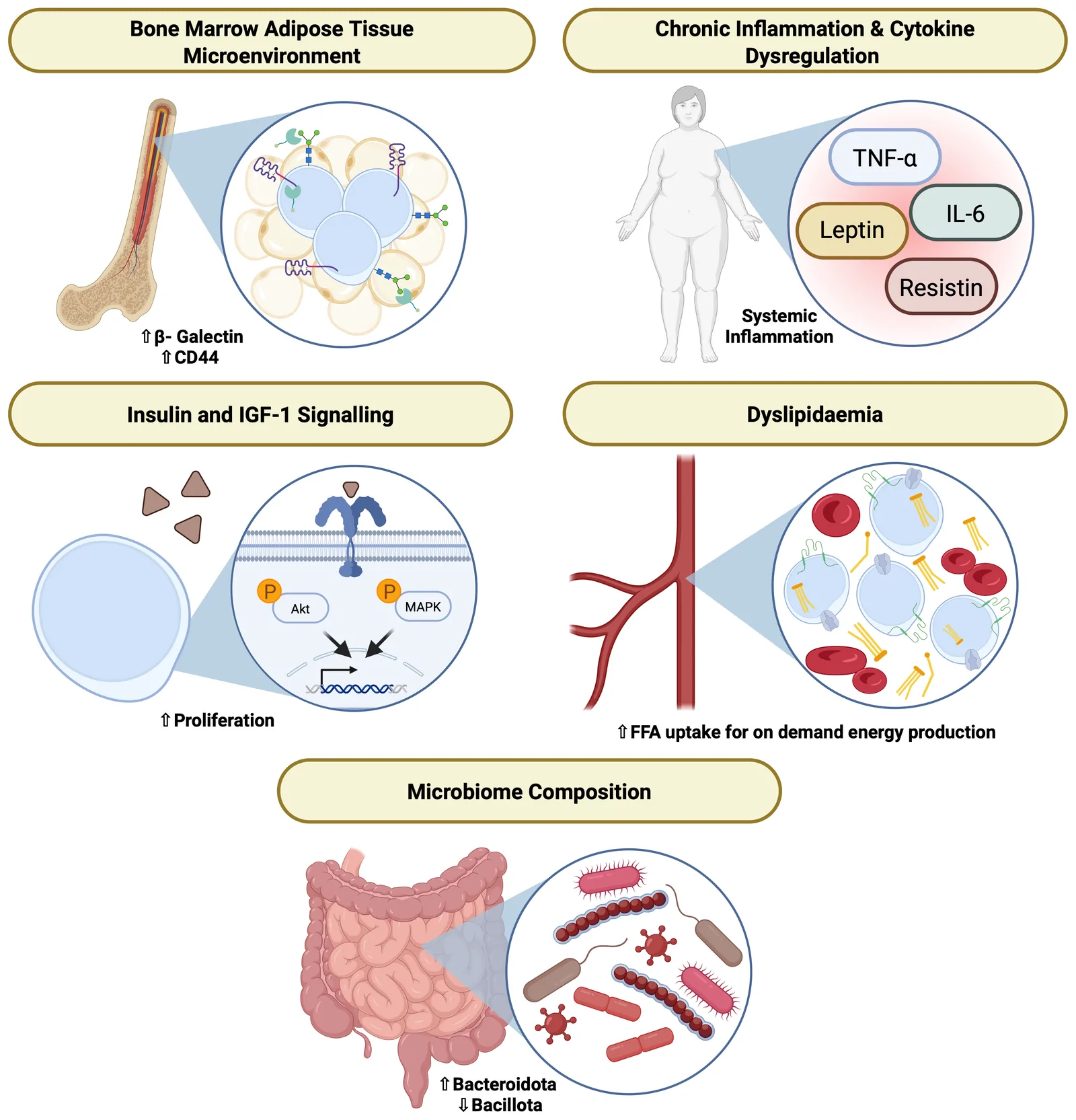
Putative mechanisms linking obesity to acute lymphoblastic progression and pathogenesis. Poor outcomes in ALL patients identified at diagnosis are hypothesized to arise due to a compounding influence of systemic inflammation; altered receptor profiles on ALL cells exposed to bone marrow adipose tissue, physiological changes in insulin-like growth factor signaling, dyslipidemia and a downregulation of commensal bacterial species.

Although a causative role for excess body weight contributing to leukemia pathophysiology and on-treatment side effects is not currently defined, major pathways linking high BMI to ALL include bone marrow adiposity (69), chronic inflammation (70), insulin signaling (71) and microbiome dysbiosis (**Figure 2**) (33, 72).

#### Bone marrow adipose tissue influences on hematopoietic cells

Adipose tissue is the body’s largest endocrine organ, known for the secretion of bioactive polypeptides (73). Adipocytes are categorized into white, beige, brown and pink, all with distinct endocrine functions. Adipocytes present within the bone marrow, referred to as bone marrow adipose tissue (BMAT), are representative of approximately 10% of the body’s total fat mass in lean individuals (74) and feature distinct metabolic and secretory characteristics. The expansion of BMAT is positively associated with the occurrence of obesity, insulin resistance and dysglycemia. Notably, BMAT adipocytes release leptin, adiponectin and adipocytokines in a pattern that varies from the secretory characteristics of visceral adipocytes (75). Hematopoiesis is thought to be directly influenced by bone marrow adipocyte secretory signals through their close proximity to the hematopoietic stem cell niche (76).

In ALL, BMAT is hypothesized to induce cellular senescence, impairing the efficacy of chemotherapy on neoplastic cells sequestered in the bone marrow, as senescent cells are cell-cycle arrested, rendering them less susceptible to many cytotoxic agents that rely on cell-cycle progression to induce DNA damage and cellular death (69, 77). Murine studies demonstrate that bone marrow niches differentially protect T-ALL cells from chemotherapy (69). Injection of human T-ALL cells into the tail bone marrow (yellow marrow; fat stores) results in low cell cycle progression, low expression of surface markers and low metabolism. Conversely, cells introduced to the thoracic bone marrow (red marrow; blood cell production) demonstrate rapid proliferation, high expression of surface markers and active cell cycle progression (69). This indicates a delay in T-ALL expansion within adipocyte-rich murine bone marrow. Moreover, T-ALL cells in the yellow BM compartment express a distinct cell-surface phenotype compared to cells in the thorax and femur, with increased CD44 expression, known to increase chemoresistance by upregulated drug efflux (78).

B-ALL murine studies have demonstrated that diet-induced obesity in C57BL/6 mice potentiates ALL progression in the presence of an intact immune system (79). *In vitro*, multiple mechanisms have been identified as possible facilitators of leukemic progression. Human B-ALL cell lines grown in adipocyte-conditioned media (ACM), compared to those maintained in standard RPMI growth medium, demonstrated significant upregulation of β-galactosidase (βGal) activity, alongside activation of pro-survival signaling pathways, including AKT, ERK and XIAP (79). Activation of these pathways is known to promote cell-cycle arrest and the induction of cellular senescence, reducing proliferative capacity and altering treatment responsiveness. Consistent with this phenotype, exposure to ACM pre-conditioned media before chemotherapy treatment was associated with a 20-50% reduction in chemotherapy-induced cell death, suggesting that adipocyte-derived factors promote a senescent, therapy-resistant state in B-ALL cells (79).

#### Chronic inflammation and associated dysregulation of cytokines

Chronic inflammation refers to systemic inflammation triggered in the absence of an acute infectious insult. It increases the risk of metabolic syndrome, cardiovascular disease, neurodegenerative disease and various oncological tumors (72, 80). Obesity is linked to chronic inflammation through the upregulation of a variety of pro-inflammatory cytokines, such as IL-6 and TNF-α (81). Obesity and serum IL-6 levels are positively correlated - one third of total circulating IL-6 is thought to originate from adipose tissue (82).

Pediatric ALL patients have distinct inflammatory profiles that share similarities with those of systemic chronic inflammation. Serum analysis of ALL patient samples at diagnosis, compared to healthy controls, reveals a significant increase in circulating levels of TNF-α, IL-6, IL-8, MCP-1, IL-12, IFN-γ, and IL-2 with a non-significant increase in IL-1β (70). Interestingly, levels of pro-inflammatory cytokines are similar in the presence and absence of fever, indicative of an ongoing inflammatory state regardless of infectious status (70).

Alongside cytokine dysregulation, obesity exerts an important impact on circulating adipokines. Adipokines collectively refer to hormonally active molecules excreted from adipose tissue. Adverse obesity-related outcomes are associated with increased circulation of leptin, resistin and visfatin along with a reduction in the adiponectin/leptin ratio(209). Adiponectin is known to modulate insulin sensitivity and has potent anti-lipotoxic effects (83),

Conversely, leptin has been shown to exert detrimental effects in chronic inflammatory diseases (83). In the context of appetite signaling, leptin signaling is mediated primarily through the long isoform of the leptin receptor, which is highly expressed in the arcuate and ventromedial nuclei of the hypothalamus. Activation of this receptor influences key neuronal populations and regulates the expression of neuropeptides involved in energy balance and appetite control (83, 84). Seminal studies in the late 1990’s demonstrated that circulating leptin levels positively correlate with adipose tissue mass in obesity, leading to the concept of leptin resistance, whereby elevated leptin fails to elicit appropriate physiological responses (85, 86). More recent work has expanded knowledge of leptin’s role beyond metabolic regulation, identifying it as an important mediator of adaptive immunometabolism, with leptin observed to enhance the proliferation of pro-inflammatory CD4^+^ cells, while reducing the proliferation of T_reg_ cells (87). Furthermore, leptin is observed to induce inflammatory mediators and promote immunosenescence of B-cells (88).

In both adult and pediatric ALL patients, decreased adiponectin concentrations are observed in association with an increase in leptin (89) and resistin levels (90). Pediatric patients with a high BMI are shown to have increased leptin levels at diagnosis, compared to healthy weight patients (91). Comparing adipokine levels at ALL diagnosis, compared to relapse, demonstrates that at relapse adiponectin appears significantly decreased, with resistin and leptin significantly increased (90). Mean levels of resistin and leptin are also reported to be positively correlated with leukemic burden, implicating dysregulated adipokine levels in ALL pathogenesis (92).

Strikingly, a *N-Myc* murine model of ALL demonstrated that intermittent fasting reduced leptin levels and inhibited the growth of B-ALL cells in the animals (93). Experimental validation in Notch-1 T-ALL mice and MLL-AF9 Acute Myeloid Leukemia (AML) mice resulted in significantly decreased T-ALL growth, however no reduction in leukemic burden in AML animals. Inhibition of disease progression in this model is attributed to upregulation of *LEPR* expression and its downstream signaling through PR/SET domain 1, promoting cellular differentiation. Lu et al. speculated that a reduction in systemic leptin as a result of fasting leads to an increase in LEPR, providing a novel therapeutic avenue for *de novo* and relapsed ALL (93). It is critical to consider, however, the differences between murine and human metabolism, with mice having a much higher mass-specific metabolic rate than humans, complicating the translational relevance of this work to a human metabolic fasting state (94).

#### Insulin and IGF-1 signaling

Insulin is responsible for the regulation of blood glucose levels, with insulin resistance resulting in type 2 diabetes (95). Obese patients frequently have relative insulin resistance and metabolic syndromes (95). Chronic hyperinsulinemia is associated with an elevated risk of solid tumors, including breast, ovarian and prostate cancers (96). In obese adolescent ALL patients, the prevalence of insulin resistance and metabolic syndrome is observed to be significantly higher compared to healthy weight patients, which may be associated with exacerbation of obesity on treatment and poorer ALL outcomes (97). Additionally, drug-induced diabetes mellitus (DIDM) is common in ALL patients, with obesity a known risk factor for the occurrence of DIDM (98).

The roles of insulin, insulin resistance, and the insulin-like growth factor 1 (IGF-1) axis are proposed to be multifaceted in ALL. Distorted IGF-1 signaling is associated with the development of aggressive/refractory disease in AML during induction chemotherapy and stem cell transplantation (99). An Egyptian cohort of pediatric ALL patients observed significantly elevated expression of IGF-1 in ALL patients at diagnosis, compared to healthy controls (71). In T-ALL, high IGF-1 receptor expression is maintained by Notch signaling (100), whereas MAPK signaling is implicated in the proliferative effect of IGF-1 on pre-B-ALL cells (101). Obese patients are often reported to have low concentrations of total IGF-1, but elevated free (active) IGF-1 due to lower expression of IGF binding proteins (102). *In vitro* supplementation of pre-B-cell cultures with IGF-1 results in a dose-dependent increase in cellular proliferation accompanied by enhancement of IGF-1R, MAPK and Akt phosphorylation (101). Furthermore, IGF-1 supplementation was observed to decrease signaling in the bone marrow microenvironment, supporting an increased resistance capacity for cells within this niche (103).

Additionally, insulin receptor substrate 1 (IRS1), together with IGF-1 signaling, has been implicated in therapeutic resistance in ALL, with alterations in IRS1 expression and phosphorylation contributing to enhanced survival signaling and reduced treatment sensitivity (104, 105). IRS1 is a gene signaling adaptor, connecting surface insulin receptors to intracellular regulation of glucose metabolism (106). CD34+ bone marrow cells express *IRS2* as the primary transcript, whereas CD3+ lymphocytes highly express *IRS1* (105). Fernandes et al. identified high levels of IRS1 protein expression in T-ALL cell lines Jurkat and MOLT4, and Burkitt’s lymphoma cell lines Raji and Namalwa, observing colocalization with β-catenin in the nucleus and cytoplasm (107). In clinical samples, comparison of peripheral blood mononuclear cells from thirteen healthy donors and forty-five adult ALL patients identified higher detection of IRS-1/β-catenin mRNA in ALL patient cells (107). The insulin receptor substrate axis appears critical in selecting targeted therapies in childhood ALL, with the axis important in determining the pro- or anti-apoptotic response to AMPK activators in human ALL cell lines (108). Furthermore, IGF1R/IRS1 inhibition, using the novel pharmacological inhibitor NT157, is observed to modulate the expression of more than 25 genes within the MAPK signaling pathway in Jurkat cells, including oncogenes and tumor suppressors (104). Additionally, NT157-mediated IRS1 inhibition blocks mTOR and AKT signaling, implicating it/this pathway as a potential therapeutic target in obese ALL patients (104).

Treatment of Ph+ SUB15 and Ph- NALM6 immortalized human B-ALL cell lines and 5 primary patient-derived samples (3 Ph- and 2 Ph+) with the ABL inhibitor GZD824 resulted in the downregulation of IRS1 expression and subsequent suppression of downstream PICK/AKT signaling, leading to the induction of apoptosis (103). Notably, the inhibitory effects of GZD824 were more pronounced in Ph- cells, indicating that its anti-leukemic activity is not solely dependent on *BCR::ABL1* inhibition and extends to *BCR::ABL1*-independent survival pathways. Consistent with *in vitro* experiments, CZD824 significantly suppressed tumor growth in Ph- xenografts, but not Ph+ xenografts. Importantly, Ye et al. demonstrated that exogenous IGF-1 was able to rescue GZD824-mediated inhibition of IRS1 signaling, further implicating the IGF/IRS axis as a potential contributor of poorer survival outcomes in obese ALL patients (103). Overall, while these studies support a biologically relevant role for the IGF-1/IRS signaling axis in ALL pathogenesis and treatment resistance, the evidence is largely derived from cell line and xenograft models and is disproportionately focused on kinase-driven subtypes, limiting the generalizability across the clinical heterogeneity of ALL.

#### Dyslipidemia

Affecting approximately 60-70% of obese individuals, dyslipidemia is a common metabolic disorder characterized by high triglycerides (TG), reduced high-density lipoprotein (HDL) levels and elevated LDL levels (109, 110).

Dyslipidemia is also a key characteristic of pediatric ALL, irrespective of weight status, due to the widespread administration of corticosteroids and asparaginase (111). The NOPHO2008 study demonstrated 99% of ALL patients demonstrated dyslipidemia at diagnosis, with a reduction in HDL levels observed in 98% of patients and mild hypertriglyceridemia in 58% of patients (111). Interestingly, LDL levels are observed to be normal in 86% of leukemic patients from this Nordic cohort.

Obese individuals are observed to have higher concentrations of circulating free fatty acids (FFAs) (112). Additionally, alterations of circulating FFA profiles are observed in acute leukemias and pre-leukemic conditions such as aplastic anemia and can indicate progression into oncogenic disease (113, 114). Free fatty acid receptor (FFAR) and transport protein expression are also implicated in a variety of solid tumors, as exogenous uptake of FFAs allows metabolic flexibility. Well-characterized mechanisms of FA signaling and uptake include CD36, fatty acid binding proteins, and fatty acid transport proteins (115). In the context of ALL, it has been pre-clinically demonstrated that murine adipocytes release FFAs when in the presence of ALL cells (116). Furthermore, these FFAs are taken up by ALL cells and stored intracellularly for on-demand energy production. The unsaturated fatty acid, oleic acid, is shown to protect cells from modest concentrations of chemotherapy, with this posited to contribute to decreased treatment efficacy in the obese patient population (116).

#### Microbiome composition

The composition of the gastrointestinal microbiome has been shown to regulate the accumulation of adipose tissue within the host and 16S rRNA gene sequencing links the obese phenotype with two dominant bacterial phyla; Bacillota and Bacteroidota (117). Overweight or obese individuals are found to have a 50% reduction in Bacteroidota, with a corresponding increase in Bacillota, compared to healthy-weight controls (33, 72). An altered Firmicutes/Bacteroidetes ratio (F/B ratio) is also emerging as a microbial signature of dysbiosis in pediatric patients with ALL. In children who develop obesity during treatment for ALL, specific microbiome perturbations are observed and hypothesized to be associated with weight gain in this context. Shotgun metagenomics identified increases in several bacterial species (e.g. *Klebsiella pneumonia*) in overweight/obese children; however, larger patient cohorts are needed to strengthen evidence (24).

Obesity, a westernized diet, excessive alcohol consumption and hyperglycemia are all proven to negatively modulate the integrity of the gastrointestinal barrier (118–120). Alterations involve the reduction of tight junctions, altered production of anti-microbial peptides and mucus layer composition (121, 122). These modifications lead to low-grade inflammation due to metabolic endotoxemia. Referring to a chronic inflammatory condition characterized by increased levels of systemic circulating lipopolysaccharides (LPS), metabolic endotoxemia exacerbates metabolic dysfunction and may contribute to leukemia pathogenesis (123). Exploration of LPS on AML pathophysiology reveals decreased apoptosis in CCK-8 cells treated with LPS *in vitro* (124). Furthermore, *in vivo* systemic LPS administration in AML mice results in increased splenomegaly, increased leukemic burden, and decreased overall survival compared to vehicle-treated animals (124).

#### Mechanistic intersections

**Figure 3:**
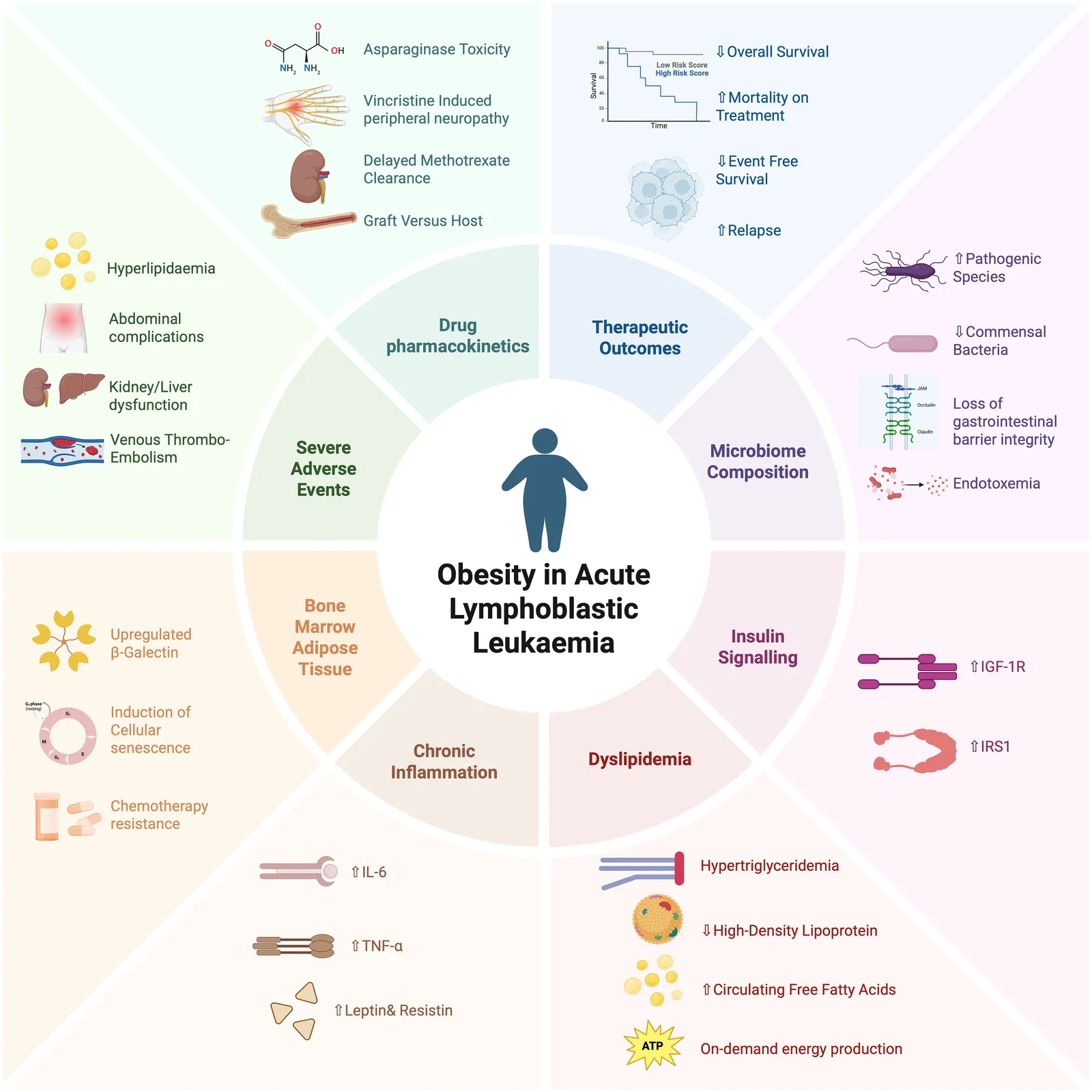
Clinical and biological observations connecting obesity and acute lymphoblastic leukemia. Obesity is hypothesised to contribute to the pathogenesis and progression of ALL through interconnected mechanisms, resulting in severe adverse events, undesirable drug pharmacokinetics, and poor therapeutic outcomes. These mechanisms include chronic inflammation, insulin resistance, dyslipidemia, microbiome composition and accumulation of bone marrow adipose tissue.

Proposed biological mechanisms associating obesity with poorer ALL outcomes converge through shared disruption of the bone marrow microenvironment and host metabolic homeostasis. Expansion of bone marrow adipose tissue increases localized lipid storage, enriching the bone marrow niche with free fatty acids, adipokines and metabolites that may be utilized as an energy source by leukaemic cells and protect them from antineoplastic stress (116, 125). Similarly, dyslipidemia contributes to additional circulating lipid substrates, supporting metabolic flexibility in leukaemic cells when they exit the bone marrow into peripheral circulation (99). Simultaneously, obesity-associated systemic inflammation may activate pro-survival signaling cascades (126), and impair normal immune surveillance (127). Insulin resistance and dysregulation of the insulin/IGF-1 pathway may further amplify these effects by promoting anabolic signaling and anti-apoptotic pathways in leukaemic cells while reinforcing chronic inflammation (74). In parallel to these physiological changes, microbial dysbiosis resulting in reduced intestinal permeability and endotoxin production further exacerbates systemic inflammatory states and cytokine production (128). Together, these intersecting processes are hypothesized to reinforce one another within an obesogenic state, resulting in a bone marrow and peripheral environment that is metabolically rich, immunologically dysregulated and permissive to leukemic survival and therapy resistance **Figure 3**.

## Conclusions

This review explores the association between obesity and acute lymphoblastic leukemia, epidemiological evidence, clinical observations and biological mechanisms that connect metabolic dysfunction to ALL outcomes. At a population level, increases in the global incidence of obesity and ALL are observed in parallel. Through clinical data, we can ascertain that obesity is strongly correlated with poorer survival outcomes in children, adolescents and young adults diagnosed with ALL. Poorer survival outcomes are attributed to an increased incidence of therapeutic toxicity resulting in treatment delays and dosing challenges. While clinical data increasingly demonstrate the influence of obesity on ALL outcomes, these observations are underpinned by a complex network of biological mechanisms. Leukemia-adipocyte crosstalk in the bone marrow microenvironment is observed to induce cellular senescence of B and T-ALL cells, resulting in a subset of chemotherapeutic-resistant malignant cells that may trigger relapse. Metabolic dysfunction also induces a chronic low-grade inflammatory profile with pronounced increases in circulating levels of IL-6 and TNF-α, acting to promote proliferation in pre-leukemic clones. Hallmark insulin signaling dysregulation in obesity is connected to leukemia, as obesity-induced elevated free IGF-1 levels act upon the characteristic increased IRS-1 on leukemic cells to upregulate the PI3K/AKT pathway. Additionally, upregulated FFAs in obesity are taken up by ALL cells to be stored for on-demand energy production.

Future clinical investigations should focus on prospective, multicenter cohorts to better define temporal and causal relationships between obesity and ALL outcomes across diverse patient populations. Global collaboration is necessary to ensure upcoming studies can be adequately cross-compared, with standardized measures of obesity, consistent reporting of survival outcomes and full transparency of treatment protocols and supportive care administered. We recommend that future work move away from BMI measurement and toward a more robust measurement of body composition and sarcopenic obesity in the ALL patient cohort. Pre-clinical research should focus on the identification and validation of metabolic biomarkers that may improve risk stratification in obese patients, and metabolically dysregulated individuals that may not physically present as classically obese. Candidate biomarkers may include measures of insulin resistance, lipid metabolism, inflammatory cytokine profiles or adipokine release. Integration of metabolic markers with pre-existing clinical variables may help to distinguish patients at an elevated risk of treatment-related toxicity, relapse and reduced survival outcomes. Additionally, precision medicine should be explored to account for obesity-related biological heterogeneity, with approaches encompassing treatment adaptation based on metabolic phenotype, pharmacokinetic and pharmacodynamic variation. Ultimately, obesity-targeted interventions should be considered as an adjunct to standard ALL therapy, to improve treatment efficacy and patient comfort during treatment. Interventions could include nutritional counselling, behavioral education, and if required, pharmacologic or metabolic therapies. Randomized prospective trials will be required to determine whether such interventions can safely improve weight trajectories, reduce significant adverse events and remove the observed survival difference between obese and healthy weight ALL patients.

Obesity should not be viewed only as a comorbidity in acute lymphoblastic leukemia, but as a biologically active modifier of disease pathophysiology and therapeutic response. Deeper mechanistic understanding may open new avenues for risk-adapted therapy and targeted interventions.
